# Issue prioritisation decisions by local politicians: the role of order effects and justification requirements

**DOI:** 10.1080/03003930.2024.2374906

**Published:** 2024-07-08

**Authors:** Amandine Lerusse

**Affiliations:** Institute of Public Administration, Leiden University, The Hague, The Netherlands

**Keywords:** Performance information, issue prioritisation, order effects, debiasing technique, local governments, survey-experiment

## Abstract

When taking issue prioritisation decisions within local governments, politicians have to process extensive performance information that includes multiple dimensions. As politicians are subject to cognitive constraints, this study advances our understanding of issue prioritisation decisions through a behavioural perspective, integrating arguments from the literature on performance information and order effects. More specifically, this study investigates how the order of performance information affects local politicians’ issue prioritisation decisions. In addition, the study examines a debiasing technique (justification requirements) to reduce potential biases within local governments. Using survey-experiment data (*n* = 1,291), this study finds that the order of the evidence does not influence politicians’ issue prioritisation decisions at the local level. Yet, local politicians pay more attention to the evidence when asked to justify their issue prioritisation decisions. This study also discusses the theoretical and practical implications of the study findings.

## Introduction

Despite seemingly offering objective means for public organisations, performance information use is subject to constraints. As key decision-makers in local governments, politicians have access to a large amount of performance data, and need to process this information to decide which issues to address and how. Yet, due to human and financial constraints, politicians can only address a limited number of policy problems and therefore have to select the policy problems that will be prioritised over the ones that will be postponed or overlooked. This decision-making process is known as issue prioritisation. It refers to decision-makers’ identification and ranking of the features of a policy domain that need a response (Jones and Baumgartner 2005). Issue prioritisation can be understood from either a vertical or horizontal perspective on decision-making (van der Voet and Lerusse 2024). In the horizontal perspective, decision-makers prioritise an issue among multiple issues (i.e., what type of public service is most relevant). In the vertical perspective, they base their prioritisation decisions solely on the performance level related to a single issue (i.e., is performance of a service below or above the aspiration level), determining whether this issue should be prioritised without directly comparing it to other issues (cf. Gavetti et al. 2012, 25). This study adopts a vertical perspective on issue prioritisation.

The process of issue prioritisation becomes more complex when performance evaluations include multiple performance dimensions (Christensen et al. 2018). Multidimensional performance assessments entail that some dimensions can be evaluated positively while others can receive a negative evaluation, leading to ambiguous performance assessments. Therefore, in a context where politicians cannot examine all performance information at the same time, the order in which performance dimensions appear in decision-making processes may impact politicians’ issue prioritisation decisions, a mechanism defined as order effects. Order effects capture ‘the tendency of people to evaluate information differently depending on the order in which the information is presented’ (Christensen and James 2022, 143).

While previous research has shown that the way information is presented impacts politicians’ preferences (Blom‐Hansen, Baekgaard, and Serritzlew 2021), the influence of order effects on politicians’ decision-making has rarely been examined in the Public Administration literature. This is surprising because politicians’ decisions determine the design, development and implementation of public policies. More specifically, order effects can influence politicians use of performance information, resulting in a biased use of performance information. According to Kahneman and Tversky (1996), cognitive biases can result in suboptimal decision-making in comparison with a normative standard.^1^ Suboptimal issue prioritisation decisions within local governments can lead to an inefficient allocation of resources and can hinder the quality of public services as some issues could be overlooked while others could be erroneously prioritised. It may decrease citizens’ satisfaction with public services and limit citizens’ uptake of public services, hindering the achievement of public policy goals.

Although recent empirical studies investigate how cognitive biases can be reduced (Cantarelli, Belle, and Belardinelli 2018; Christensen and Moynihan 2020; Nagtegaal et al. 2020), most studies exclusively focus on identifying cognitive biases rather than actively testing solutions to mitigate or eliminate them (Bhanot and Linos 2020). The scarcity of debiasing studies can be attributed to the idea that ‘cognitive biases are robust and debiasing is notoriously difficult’ (Nagtegaal et al. 2020, 566). Moreover, debiasing studies in Public Administration research indicate that some debiasing techniques tend to reinforce decision-makers’ cognitive biases (Christensen and Moynihan 2020). This study seeks to contribute to the debiasing literature by testing a debiasing technique among politicians and investigates how justification requirements can lead to more accurate decisions.

This study examines the extent to which order effects influence politicians’ issue prioritisation decisions and the extent to which potential cognitive biases can be reduced via a debiasing technique (justification requirements). This study first hypothesises that politicians are prone to order effects when making issue prioritisation decisions. Second, decision-makers who provide justifications for their decisions tend to pay more attention to the evidence and to make more accurate decisions (Lerner and Tetlock 1999; Tetlock 1983, 1992). Based on these insights, this study outlines two additional hypotheses. First, it hypothesises that politicians pay greater attention to the evidence when asked to justify their issue prioritisation decision. Second, it stipulates that politicians’ tendency to be prone to order effects is reduced when asked to justify their issue prioritisation decision. The hypotheses are tested using a survey-experiment among elected politicians in Belgian local governments (*n* = 1,291). The empirical findings show that politicians are not prone to order effects when making issue prioritisation decisions. The results also indicate that politicians pay more attention to the evidence when asked to justify their issue prioritisation decisions, but politicians’ attention level does not influence their issue prioritisation decisions.

The contribution of this study to performance information research is threefold. First, although decision-makers have cognitive constraints (Simon 1971), the role of cognitive biases on politicians’ issue prioritisation decisions has not yet been examined. By applying a behavioural perspective to investigate issue prioritisation decisions, this study advances knowledge on politicians’ issue prioritisation. Second, the role of order effects on decision-making behaviour is one of the most widely researched behavioural phenomena (Bond et al. 2007), but its impact on politicians’ decision-making behaviour remains unexplored. This study therefore sheds light on the influence of order effects on performance information use and contributes to the emergent Behavioural Public Performance approach. This perspectives aims at integrating behavioural insights into performance management (James et al. 2020). Finally, this study contributes to the nascent literature on debiasing by examining how potential cognitive biases can be reduced through justification requirements.

## Theoretical framework and hypotheses

### Issue prioritization decisions

Issue prioritisation decisions are intrinsically linked to the degree of performance of the policy domain. Behavioural theory states that decision-makers use aspiration levels as reference points against which they evaluate performance (Greve 2003). The theory further posits that decision-makers tend to prioritise issues identified as performing below an aspiration level (Cyert and March 1992; March and Alexander Simon 1958), while issues that perform above an aspiration level are less likely to be addressed. In line with this theoretical assumption, Public Administration studies find that public organisations tend to focus on issues with low performance (Hansen and Aaes Nielsen 2022; Holm 2018; Hong 2019).

Politicians have to address complex and multifaceted issues that are increasingly described by multiple performance dimensions (Christensen et al. 2018). Due to the multidimensionality of performance information, the sequence of evidence appears in a specific order. Politicians are, therefore, not always able to simultaneously assess all performance dimensions (Audia and Greve 2020). As the literature on order effects shows that decision-makers are influenced by the order of evidence (Hogarth and Einhorn 1992), this study argues that the order in which performance information is examined may affect politicians’ issue prioritisation decisions. The issues that need to be prioritised could therefore be overlooked or postponed while some issues could be erroneously prioritised.

### Issue prioritization decisions and order effects

The literature identifies two different types of order effects: a primacy effect (Tetlock 1983) and a recency effect (Mayo and Crockett 1964). The primacy effect implies that earlier pieces of evidence or the starting point of a decision-making process have a disproportionate influence on the outcome of an evaluation process. In contrast, the recency effect occurs when the outcome of the decision-making process is disproportionally affected by later pieces of evidence (Bond et al. 2007). While the primacy effect is ‘among the most widely observed phenomena in basic and applied behavioral research’ (Bond et al. 2007, 241), the recency effect is less prominent in decision-making tasks (Hogarth and Einhorn 1992). Moreover, the literature has extensively developed the theoretical mechanisms behind the primacy effect, with comparatively less elaboration on the theoretical mechanisms of the recency effect. Driven by better articulated theoretical mechanisms and larger empirical evidence, this study states that the primacy effect is more likely to influence decision-makers’ issue prioritisation decisions. It therefore elaborates on the theoretical mechanisms behind the primacy effect.

The first mechanism refers to ‘*biased search from memory*’ (Bond et al. 2007, 241). Decision-makers tend to rely on initial assessment to make subsequent evaluations due to anchoring which refers to the propensity to estimate unknown quantities based on an initial value (Kahneman 2011), opinions, or beliefs (Bond et al. 2007). For order effects, the initial evidence can therefore be considered the anchor against which decision-makers build their final evaluation by accessing anchor-consistent information in their memory. For instance, positive anchors lead to more positive evaluations, while negative anchors lead to more negative evaluations (Bond et al. 2007).

The second mechanism relates to ‘*biased search from the environment’* (Bond et al. 2007, 241). Decision-makers, who base an opinion on earlier pieces of evidence, tend to search for subsequent information that aligns with their initial impressions while avoiding subsequent information that diverges from their initial opinion (Bond et al. 2007; Tetlock 1983). This mechanism relates to the theory of motivated reasoning, where decision-makers tend to downplay or disregard the evidence that is not in line with their initial beliefs to deal with the uncomfortable state of cognitive dissonance (Kunda 1990; Milton and Charles 2000; Taber and Lodge 2006).

The last theoretical mechanism focuses on ‘*biased weighting of information*’ (Bond et al. 2007, 242). Decision-makers subjectively encode new evidence that is systematically compared to a reference point. The evidence is then weighted and absorbed in decision-makers’ prior evaluations of the evidence. The overweighting or underweighting of the evidence compared to the reference point depends on several factors, such as the number of evidence pieces that decision-makers have to evaluate or the complexity of the evidence. The primacy effect is often associated with longer and more complex tasks. In contrast, the recency effect is more likely to occur when the evaluation process is short and simple (Bond et al. 2007). Decision-makers may also be less sensitive to additional evidence and could consequently weigh them less significantly (Hogarth and Einhorn 1992), indicating a primacy effect.

While research on primacy effects states that earlier evidence in the performance sequence carries more weight in decision-making outcomes, the behavioural theory of the firm posits that issues below an aspiration level should be prioritised and that issues above an aspiration level should not be prioritised (Cyert and March 1992; March and Alexander Simon 1958). This study connects the behavioural theory of the firm with research on primacy effects. More specifically, it states that if negative evidence precedes positive evidence in the performance sequence, politicians will prioritise the issue; however, they will not prioritise the issue when the positive evidence precedes the negative evidence in the performance sequence. Despite its relevance and influence on decision-makers’ behaviour, the investigation of primacy effects in the Public Administration literature, particularly concerning politicians’ issue prioritisation, has been hitherto absent. Yet, Public Administration scholars have shown that politicians are also influenced by how performance information is presented (Sheffer et al. 2018), indicating that they may not be immune to primacy effects when using performance information data. If politicians are prone to primacy effects, issues that require a response may be overlooked or postponed, while issues that do not need to be addressed may be prioritised, impacting policymaking. Based on these observations, this study formulates the following hypothesis:

H_1_: Politicians are more likely to prioritise issues based on earlier performance information rather than later information.

## Issue prioritisation decisions and justification requirements

Issue prioritisation decisions often require politicians to provide justifications. The objectives of justification requirements are twofold: to clarify politicians’ use of performance information while intending to hold politicians accountable for their decisions (Belardinelli et al. 2018). Justification is therefore perceived as a type of accountability mechanism that is considered an omnipresent characteristic of decision-making in public organisations (Voet Joris van and Rimkutė 2022). Accountability is defined as a relationship ‘between an actor and a forum, in which the actor has an obligation to justify his or her conduct to the forum’ (Aleksovska 2021, 707), and is perceived as an instrument for democratic control (Aleksovska, Schillemans, and Grimmelikhuijsen 2019).

Tetlock (1992, 337) states that ‘accountability is a critical rule and a norm enforcement mechanism – the social psychological link between individual decision-makers on the one hand and social systems on the other’. Accountability motivates careful and complex assessment of the evidence because decision-makers seek approval and respect to secure and improve their identity, social image, and self-image and to obtain power and wealth (Tetlock 1992). Accountability also implies that decision-makers who provide satisfactory justifications will experience positive consequences (Lerner and Tetlock 1999). Yet, the influence of accountability depends on whether decision-makers know that they have to justify their choice before (pre-decisional accountability) or after (post-decisional accountability) seeing the evidence that they need to assess (Tetlock 1983). While pre-decisional accountability eliminated primacy effects as it stimulates decision-makers to attentively and carefully process the evidence, post-decisional accountability did not have any effects (Tetlock 1983). These theoretical insights suggest that requiring politicians to justify their issue prioritisation decisions could be a meaningful debiasing technique.

Past accountability studies show that making decision-makers accountable for their choice increases their search efforts (Lee et al. 1999), their information remembering (de Dreu et al. 2006), and their processing of information (Roch 2006). Moreover, decision-makers, who were held accountable, were also found to take longer to make decisions (Lee et al. 1999). However, Public Administration studies examining the role of justification requirements on politicians’ choices have led to mixed results. Belardinelli et al. (2018) find that including justification requirements did not improve public managers’ performance assessments. Similarly, Julian and Moynihan (2020) show that justification requirements increase politicians’ likelihood to engage in motivated reasoning. Requiring decision-makers to justify their decisions may strengthen their intuitions due to confirmation bias, reinforcing the existing cognitive bias.

In contrast, the influence of justification requirements on attention has demonstrated positive results. van der Voet (2023) shows that attention can be measured via politicians’ reading time and suggests that public officials, who spend more time reading the evidence, assess it more carefully. Julian and Moynihan (2020) find that requiring politicians to justify their decisions increased their attention to the evidence by 21%.

Drawing on insights from accountability research in social psychology and recent evidence in Public Administration literature, this study states that justification requirements can be used as a debiasing technique. More specifically, this study argues that justification requirements can influence politicians’ issue prioritisation decisions in two different ways. On the one hand, justification requirements can increase politicians’ attention when processing evidence. On the other hand, it can lead to more accurate decisions by reducing potential order effects. This study, therefore, formulates the following hypotheses:

H_2_: Politicians process evidence with greater attention when asked to justify their issue prioritisation decisions.

H_3_: Politicians’ tendency to be prone to order effects is reduced when asked to justify their issue prioritisation decisions.

## Methodological approach

The way performance information is presented may be endogenous to politicians’ perceptions of public service performance, indicating that the supplier of performance information may anticipate their response by designing performance data in specific ways (Christensen and James 2022). Therefore, the causal link between order effects and politicians’ issue prioritisation decisions is difficult to establish with observational data. This study uses a randomised survey-experiment where politicians are randomly allocated to different experimental groups to get closer to causal claims.

### Research setting – primary school education at the local level

The randomised survey-experiment focuses on the municipal level in Belgium. In Belgium, local governments include a legislative body (the municipal council) and an executive body (the municipal college). The municipal council comprises the mayor, the aldermen, and the municipal councillors. It mainly makes decisions regarding the design and implementation of policy programmes. The municipal college, composed of the mayor and the aldermen, executes the policy decisions the municipal council has approved. The municipal residents are responsible for electing local politicians who have a mandate of six years (Deschouwer 2012). Belgian local politicians have substantial autonomy and considerable discretion concerning the design and implementation of local public services such as waste collection, road maintenance, or primary school education (Deschouwer 2012). Their extensive range of competencies gives them ‘a real and visible impact on the daily life of the citizens’ (Deschouwer 2012, 178). Given local politicians’ direct impact on the lives of their population, this study argues that it is essential to better understand the influence of order effects on local politicians’ issue prioritisation decisions.

Among the numerous public services that are delivered at the local level, this study concentrates on public primary school education. The performance of Belgian public primary schools has been falling in recent years, as illustrated by the declining results of the certificate of basic studies, obtained by primary school pupils at the end of primary school. As a result, concerns about the performance of Belgian pupils have grown among public authorities and citizens. Yet, school performance is increasingly being assessed via multiple performance dimensions (Christensen and James 2022), thereby leading to ambiguous performance assessments. As local politicians oversee public primary school education and may regularly face ambiguous performance assessments in this policy domain, it is essential to investigate the extent to which order effects can influence local politicians’ issue prioritisation decisions concerning public primary school education.

### Experimental design

This study extensively draws on the experimental design developed by Christensen and James (2022). Their research examines the role of order effects on citizens’ evaluation of school performance and shows that order effects impact citizens’ willingness to use the school, but not citizens’ overall favourability with the school. The present study builds on Christensen and James (2022)’s work by focusing on a sample of politicians – rather than citizens – in a distinct research setting. This study also uses a different dependent variable by focusing on politicians’ issue prioritisation decisions. Moreover, compared to Christensen and James (2022)’s research, this study incorporates an additional experimental branch which is designed to test the second and third hypotheses about justification requirements. This study therefore brings further empirical evidence on the role of order effects in public administration research, a topic that has hitherto been neglected in the literature.

**Figure 1.**
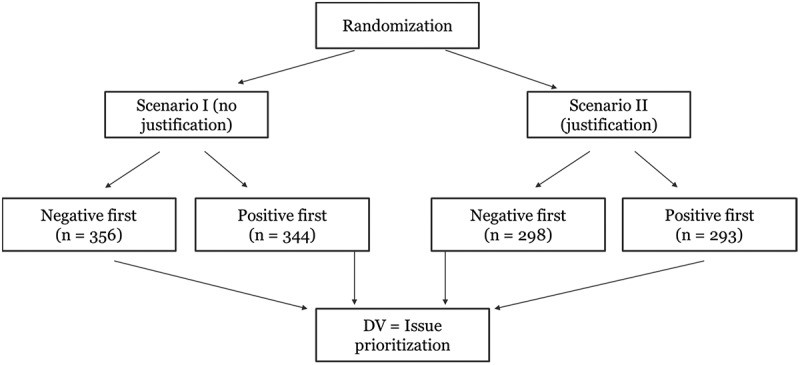
Experimental design and flow.

This study applies a randomised survey-experiment (2 × 2 factorial design) about an issue prioritisation task among local politicians. It uses a between-subject design where participants are randomly allocated to one of four experimental groups. Each participant, therefore, has to read one performance task. The between-subject design is cognitively less demanding for the participants and minimises learning effects compared to within-subject designs. The outcome variable, issue prioritisation, is measured with the following question: Considering all the previous information, to what extent do you think it is necessary to prioritise the improvement of primary education in this municipality (on a scale from ‘0 – not necessary to prioritize’ to ‘100 – very necessary to prioritize’)? Figure 1 presents the experimental design and flow. All the participants had to give their informed consent before participating in the survey.

**Figure 2.**
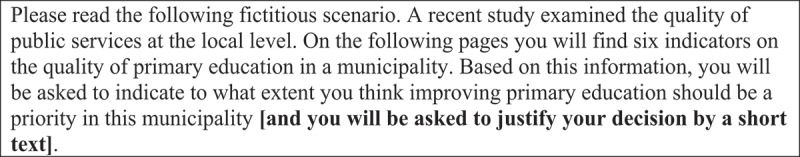
Fictitious scenario.

Participants are randomly assigned to either the neutral scenario (no justification requirements) or the scenario asking them to justify their decision (justification requirements). Decision-makers pay more attention to the evidence when the accountability mechanism is placed at the beginning of the decision-making task (Tetlock 1983). Thus, participants were informed in the introductory scenario whether they would have to justify their decision at the end of the task. Participants, who had to justify their reasoning, were asked to fill out a text box following their issue prioritisation decision. Fictitious primary schools were presented to participants to ensure they did not associate the performance data displayed with existing primary schools. Participants, therefore, either received the neutral scenario or the scenario including the accountability mechanism (see Figure 2). This study tests the second and third hypotheses by randomising participants into the first or second scenario. While the second hypothesis states that politicians process evidence with greater attention when asked to justify their issue prioritisation decisions, the third hypothesis posits that politicians’ tendency to be prone to primacy effects is reduced when asked to justify their issue prioritisation decisions. Building on van der Voet (2023)’s research, this study operationalises attention as politicians’ time reading the performance sequence.

Randomly allocated to the experimental groups with the negative evidence first or positive evidence first conditions. While in the first condition, the negative performance information is placed at the beginning of the sequence, and the positive performance information is placed later in the sequence, in the second condition, it is the other way around. Drawing from the behavioural theory of the firm, decision-makers are more likely to prioritise issues that are below an aspiration level and yield negative performance information (Greve 2003), regardless of the order in which the evidence is presented. This study expects that the evidence at the beginning of the performance sequence – irrespective of whether this evidence is positive or negative – will influence the evaluation of the remaining attributes. It indicates that, when negative evidence is positioned first in the performance sequence, it should lead to more prioritisation whereas, while when positive evidence is placed first, it should lead to less prioritisation.

**Figure 3.**
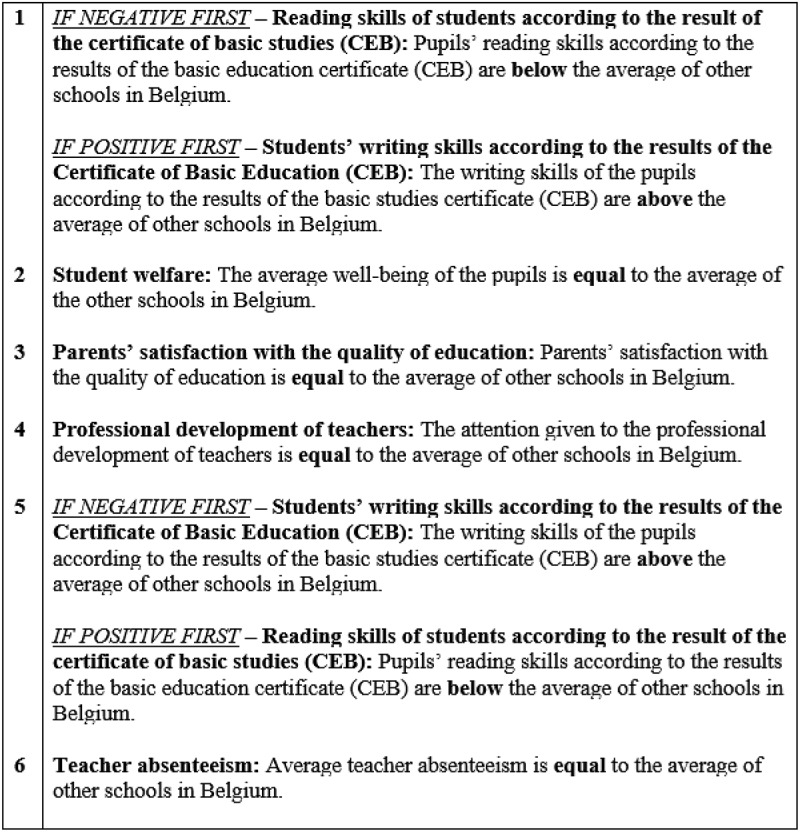
Experimental material.

This study explores the influence of order effects on politicians’ issue prioritisation decisions, employing an experimental design previously implemented by studies on order effects (Bond et al. 2007; Christensen and James 2022). The study shows six different performance indicators to the participants. These performance indicators are commonly used in education assessments. Each performance indicator is presented on a different page to the participants. One performance indicator is systematically negative (pupils’ reading skills), and one is systematically positive (pupils’ writing skills). Following previous experimental designs investigating order effects, the four other items are intentionally designed to convey neutral performance information. The performance assessment task is ambiguous because it includes a positive and a negative item. While the negative performance indicator (pupils’ reading skills) is placed at the beginning of the sequence in the first condition, it is placed later (5^th^ position) in the sequence in the second condition. The neutral items remain in the same position for all the participants. Figure 3 shows the experimental material.

### Sample and data collection

The randomised survey-experiment was distributed among 13,035 Belgian elected local politicians (mayors, aldermen, and municipal councillors). Their contact details were collected via an online database and manually. The questionnaire was sent electronically via Qualtrics to their professional email addresses. The data collection started on the 22^nd^ of June 2022 and ended on the 30^th^ of September 2022. Two reminders were sent to the local politicians. In total, 1,291 local politicians participated in the survey-experiment, leading to a response rate of 9.9%. The sample size calculation indicated that the survey-experiment needed a minimum of 1,012 study participants (α = .05; power = .80; t-tests, means, difference between two independent means), with predicted effect sizes of *f* = .25. The sample size requirement is therefore fulfilled.

Most local politicians identify with the male gender (Appendix A). They have an average age of approximately 52 years old and predominantly possess a university degree (81.6%). Forty percent of the local politicians are currently doing their first political mandate at the local level and most of the local politicians come from the Flemish region. Appendix B compares the demographic (function, gender and region) and municipal (population size and median income) characteristics of the overall population and the sample. It shows that the sample seems to be an adequate approximation of the overall population. Although politicians from Brussels tend to be slightly overrepresented in the sample, this overrepresentation should not influence the findings as politicians from the three regions perform the same activities and responsibilities. The sample also contains slightly more executive politicians compared to legislative politicians. As executive and legislative politicians have different functions, their issue prioritisation decisions may be different. Separate analyses were therefore conducted as robustness checks, and show that the empirical conclusions remain identical for both executive and legislative politicians.

## Results

**Figure 4.**
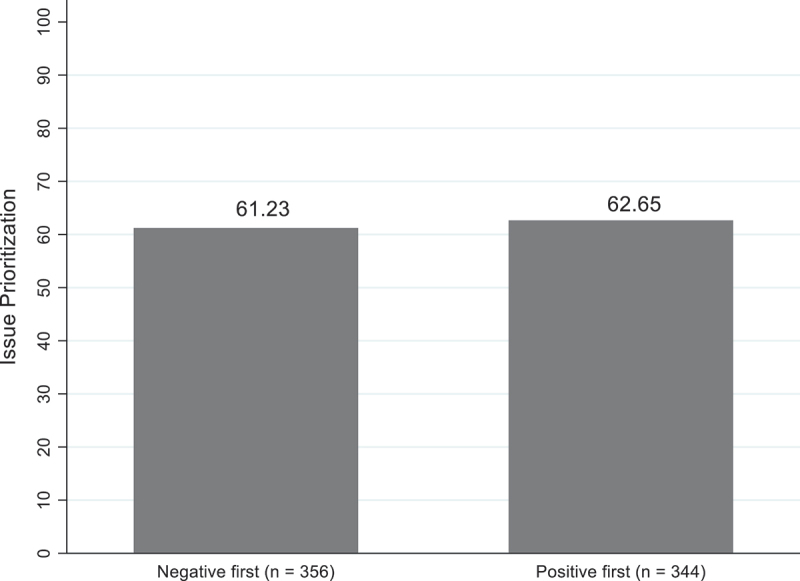
Politicians’ issue prioritization decision and order effects (*n* = 700).

Before testing the hypotheses, this study first conducts a balance test to investigate whether there are significant differences between the experimental groups concerning politicians’ individual characteristics and municipal characteristics. Appendix C shows that the experimental groups are balanced and that the randomisation process is successful. The first hypothesis (H_1_) states that politicians to prioritise issues based on earlier performance information rather than later information. To test this hypothesis, this study compares politicians’ issue prioritisation when presented with negative evidence first to politicians’ issue prioritisation when presented with positive evidence first using a t-test. The results, displayed in Figure 4, indicate that the issue prioritisation decision of politicians who are presented with negative evidence first (*M* = 61.23, *SD* = 19.90) does not significantly differ from that of politicians presented with positive evidence first (*M* = 62.65, *SD* = 19.88), *t*(700) = −.943, *p* > .1). In contrast with what was established in the literature, this study finds that order effects do not influence politicians’ issue prioritisation decisions.

Two-sample t-test were performed to test whether they are significant differences between the negative evidence first – positive evidence first – condition with respect to politicians’ issue prioritisation decision.

**Figure 5.**
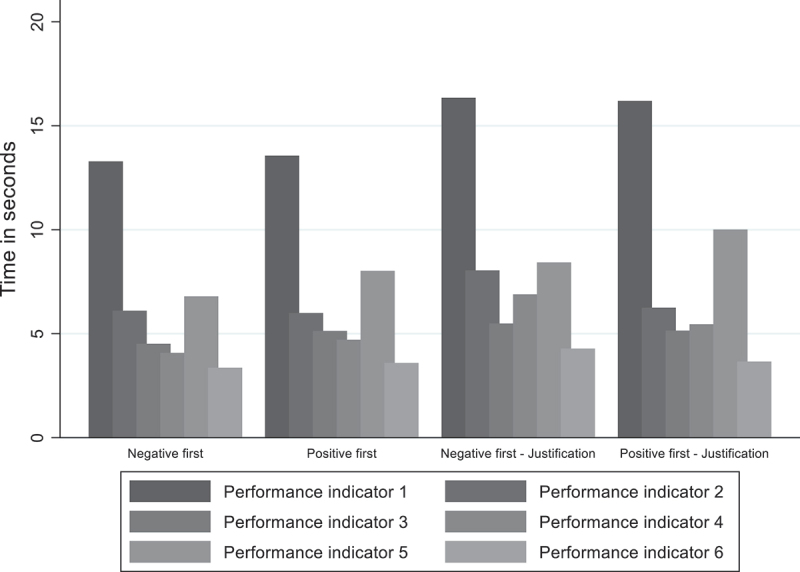
Politicians’ average time spent per performance indicator.

With regards to the second hypothesis (H_2_) stating that politicians process evidence with greater attention when asked to justify their issue prioritisation decision, this study first considers politicians’ overall attention by assessing their processing time of the evidence (van der Voet 2023). Several observations can be made by looking at the average time spent per performance indicator (Figure 5). First, it seems that politicians pay more attention to the first and fifth performance indicators in all the experimental groups, suggesting that they dedicate more time to negative and positive performance indicators. Second, politicians tend to pay more attention to the first performance indicator compared to the other performance indicators in the sequence. Third, politicians also appear to pay even greater attention to the negative and positive performance indicators in the experimental groups where they have to justify their issue prioritisation decision. These observations suggest that examining the role of justification requirements on politicians’ attention is relevant even if they are not prone to order effects. A final observation shows that politicians do not pay more attention to negative (*M* = 11.84, *SD* = 26.80) compared to positive (*M* = 11.11, *SD* = 22.24, *t*(1,291) = −.77, *p* > .1) indicators.

**Figure 6.**
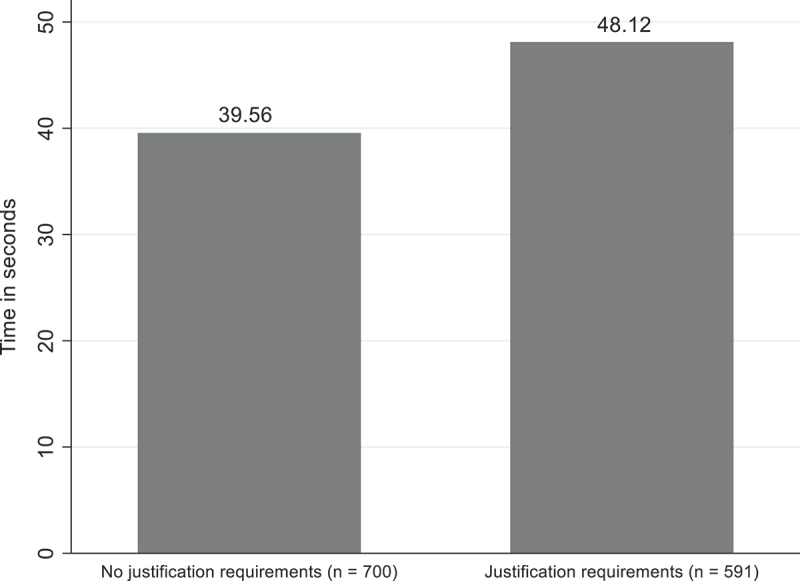
Politicians’ attention without and with justification requirements (*n* = 1,291).

This study uses a t-test to examine whether there are significant differences in attention allocation between politicians who were required to justify their issue prioritisation decision and those who were not required to justify their issue prioritisation decision (H_2_). The results indicate that politicians’ attention to the evidence in the experimental group without justification requirements (*M* = 39.56, *SD* = 28.48) is significantly lower compared to politicians’ attention to the evidence in the experimental group with justification requirements (*M* = 48.12, *SD* = 51.75), *t*(1,291) = −3.75, *p* < .001); (Figure 6). Politicians who did not have to justify their issue prioritisation decision spent on average 39.56 seconds on the decision-making task, while politicians who had to justify their issue prioritisation decision spent on average 48.12 seconds on the decision-making task. The results indicate that when politicians have to justify their issue prioritisation decision, the time they take to process the evidence increases by 21.6%.

Two-sample t-tests were performed to test whether they are significant differences between the condition without and with justification requirements with respect to politicians’ attention.

**Figure 7.**
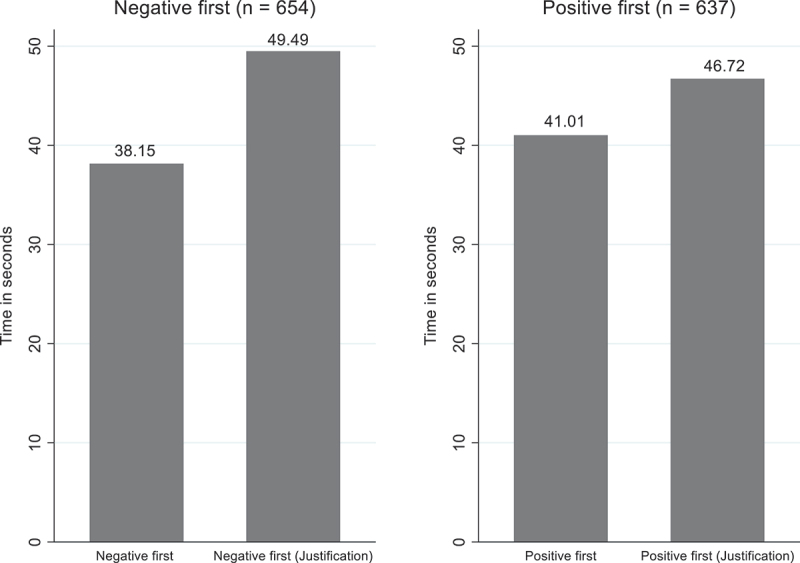
Politicians’ attention in the negative first and positive first groups (*n* = 1,291).

This study also examines the role of justification requirements within the negative evidence first and positive evidence first conditions. To do so, it compares, with a t-test, politicians who are randomly allocated to the negative evidence first condition without justification requirements to the ones who are randomly allocated to the negative evidence first condition with justification requirements. The left-hand side panel shows that the attention to the evidence of politicians who are presented with negative evidence first without justification requirements (*M* = 38.15, *SD* = 29.63) is significantly lower than that of politicians presented with negative evidence first with justification requirements (*M* = 49.49, *SD* = 51.92), *t*(654) = −3.50, *p* < .001); (Figure 7). More specifically, the results indicate that, on average, politicians who were not required to justify their issue prioritisation decision spent 38.15 seconds on the decision-making task. In contrast, politicians who are required to justify their issue prioritisation decision spent, on average, 49.49 seconds on the decision-making task, indicating an increase of 29.7% in the time spent to process the evidence when politicians have to justify their issue prioritisation decisions.

Two-sample t-test were performed to test whether they are significant differences between the negative evidence first – positive evidence first – condition without and with justification requirements with respect to politicians’ attention.

The right-hand side panel indicates that politicians who are presented with positive evidence first without justification requirements tend to significantly spend less time on the task (*M* = 41.01, *SD* = 27.21) compared to politicians who are presented with positive evidence first with justification requirements (*M* = 46.72, *SD* = 51.62), *t*(637) = −1.78, *p* < .1). However, politicians’ time spent on the task increases by 13.9% in the positive evidence first condition with justification requirements, suggesting that this effect is weaker than the effect observed for the differences between the negative evidence first conditions with and without justification requirements. When comparing the negative evidence first condition and the positive evidence first condition without justification requirements, politicians have higher attention towards evidence in the positive evidence first condition. Yet, this relationship is reversed when justification requirements are implemented. The interaction effect between ordering and justification requirements on politicians’ attention is therefore explored in Appendix D, but the results show that the interaction term is not statistically significant. Overall, the empirical results show that politicians who have to justify their issue prioritisation decision tend to spend more attention on the evidence than those who do not have to justify their issue prioritisation decision, supporting hypothesis 2 (H_2_).

The third hypothesis (H_3_) states that politicians’ tendency to be prone to order effects is reduced when asked to justify their issue prioritisation decision. The study has shown that politicians pay more attention to the evidence when required to justify their issue prioritisation decision (H_2_), suggesting that adding justification requirements to the decision-making task may influence politicians’ issue prioritisation decision. The results of the t-test show no significant differences in politicians’ issue prioritisation for both experimental conditions with justification requirements and without justification requirements (see Appendix E). This study also conducts a regression model with an interaction term (see Appendix F), confirming the results of the t-test. Hypothesis 3 (H_3_) is therefore not supported.

## Discussion & conclusion

This study investigates the extent to which order effects influence politicians’ issue prioritisation decisions and how justification requirements can be used as debiasing technique in local governments. This study takes a behavioural approach and conducts a survey-experiment, advancing our understanding of issue prioritisation at the individual decision-makers level.

This study hypothesises (H_1_) that politicians to prioritise issues based on earlier performance information rather than later information. However, in contrast with the order effects literature (Bond et al. 2007; Tetlock 1992), this study observes that politicians do not seem to be influenced by the order of the evidence when making issue prioritisation decisions (H_1_). This finding partly contradicts Christensen and James (2022)’s study findings. In their study, they examine the role of order effects on (1) citizens’ overall favourability with the school and (2) citizens’ willingness to use the school. While they find that citizens are prone to recency effects with regards to their willingness to use the school, they also show that citizens are not affected by the order of the evidence when assessing the favourability of the school. Their study findings therefore suggest that the influence of order effects depends on the outcome that is studied. These outcomes are also measured on different scales. While citizens’ willingness to use the school is measured on a binary outcome (send the child or do not send the child), citizens’ overall favourability with the school is measured on a continuous scale (‘1 – very strongly disagree’ to ‘9 – very strongly agree’), leading to more nuanced responses. The current study uses a different dependent variable – politicians’ issue prioritisation – and measures it on a continuous scale (‘0 – not necessary to prioritize’ to ‘100 – very necessary to prioritize’) rather than on a binary scale. One could therefore argue that the influence of order effects may depend on the outcome and its measurement.

The non-significant result of the present study may be related to the type of performance information use. Performance information use can be divided into two distinct situations that are referred to as ex-ante and ex-post performance information use. While ex-post performance information use refers to decision-makers’ evaluations of past performances, ex-ante performance information use refers to decision-makers’ definition of organisational objectives or allocation of resources and efforts (Belardinelli et al. 2018). Issue prioritisation decisions are considered an ex-ante performance information use because decision-makers rely on evidence to allocate resources by prioritising some issues over others. In their research, Belardinelli et al. (2018) observe that public managers are more prone to cognitive biases under ex-post performance information use. They posit that public managers who use evidence ex-ante ‘tend to explore possibilities and alternatives, probably including more elements and paying more attention in their decisions’, thereby decreasing their reliance on simple heuristics (Belardinelli et al. 2018, 844). This study findings support their empirical results and suggest that the impact of cognitive biases on politicians’ decision-making may depend on the type of performance information use. Yet, this assumption may only be valid for public officials as Christensen and James (2022) show that citizens are impacted by recency effects in ex-ante performance information use. The present study provides additional evidence on the role of cognitive biases on performance information use in local governments. It also contributes to the emergent Behavioural Public Performance approach that aims at integrating behavioural insights into performance management (James et al. 2020).

This study also aims to examine the extent to which justification requirements can be implemented as a debiasing technique among politicians. In line with previous research (Lee et al. 1999), this study finds that politicians who have to justify their issue prioritisation decisions spent, on average, 21.8% more time on the decision-making task (H_2_). This result is very similar to Christensen & Moynihan’s’ (2020)s study, which found an increase in attention of 21% for the politicians who were required to justify their assessment. Although the degree of attention does not influence politicians’ issue prioritisation decisions (H_3_), this result shows that justification requirements have the intended effect as politicians’ attention to the evidence has substantially increased (H_2_).

The conclusions provide relevant insights for local governments. Previous studies have shown that Belgian politicians’ use of evidence within local governments is biased (Lerusse and van de Walle 2022a, 2022b), questioning the effective use of performance information at the local level. Biased use of evidence is believed to impact local governance as it may lead to an inefficient allocation of local government resources, a decrease in citizens’ satisfaction and citizens’ uptake of public services, thereby impeding the realisation of public policy goals. However, this study indicates that local politicians’ use of performance information within local governments can still lead to efficient and unbiased decisions, adding nuance to the discussion about the value of using evidence within local governments. This non-significant result is also good news for the functioning of democracy within local governments. In their study, Blom‐Hansen et al. (2021) show that public managers are inclined to use performance information to influence political decisions. Yet, as politicians’ issue prioritisation decisions do not appear to be sensitive to order effects, altering the order of the evidence has limited potential to affect their issue prioritisation decisions. This conclusion decreases concerns regarding the influence of information provision on the outcome of local public policies. With regards to different governance levels, political elites in higher government levels should be less prone to biases. They tend to have more resources and support staff, potentially enabling more accurate decisions (Vis 2019). Yet, comparative work on the likelihood of politicians from different government levels to be prone to cognitive biases remains to be conducted.

The empirical results also provide relevant contributions to practice. The literature states that decision-makers do not have the information-processing abilities to process large amounts of performance data (Simon 1971). However, the results contradict this theoretical assumption and suggest that politicians are capable of processing complex performance information when taking issue prioritisation decisions at the local level. The results also show that justification requirements can be a powerful tool to increase politicians’ attention. They could therefore be used by actors who want politicians to pay more attention to the evidence during decision-making processes.

Despite its contributions to research and practice, this study has some limitations. The first limitation is tied to the experimental design of the study. The experimental design includes four experimental arms. While the first and second experimental arms did not include an accountability mechanism, the third and fourth experimental arms require politicians to justify their issue prioritisation decisions and are, therefore, cognitively more demanding. Consequently, survey participants were more likely to drop out of the survey-experiment when they had to justify their decisions (see Appendix G), possibly leading to attrition bias. Yet, the four experimental arms remain statistically balanced (see Appendix C), decreasing the risk of attrition bias.

The second limitation relates to the generalisability of the empirical findings. This study exclusively focuses on primary school education at the local level in Belgium, thereby limiting the generalisability of the study findings to this specific governance level, country, and public sector. Primary school education may also be considered a very sensitive public service, and politicians may be more likely to prioritise it. Moreover, pressures from citizens to address issues within the educational system tend to be more prominent than other types of public services, such as waste collection or road maintenance.

The third limitation concerns the measure of issue prioritisation. Following a vertical conceptualisation of issue prioritisation, the outcome variable is measured via a cardinal approach (participants assign a cardinal value to their issue prioritisation preferences). However, the measure also implicitly requires participants to prioritise primary school education against other issues, thereby implicitly incorporating an ordinal scale. This study cannot therefore specify the issues against which primary school education has been prioritised. Yet, the measurement of the outcome variable should not affect the empirical conclusions due to randomisation.

The study findings provide interesting avenues for future studies. Future research should examine the influence of order effects on different outcomes as well as various scales to have a better understanding of the differences between this study finding and Christensen and James (2022)’s conclusions. Building on the idea that public officials may be less prone to cognitive biases in ex-ante performance information use compared to citizens, this study recommends further research to compare the impact of cognitive biases on the decision-making of both public officials (including public managers and politicians) and citizens in different types of performance information use. As this study adopts a vertical perspective on issue prioritisation, it would be necessary to replicate this study from a horizontal perspective, by examining the influence of order effects when decision-makers take prioritisation decisions based on several issues. It also remains essential to examine the role of order effects on issue prioritisation decisions in other public services at the local level, especially in technical services, which may be less salient and, therefore, less likely to be prioritised by politicians. Moreover, technical services such as road maintenance or waste collection have received less attention in the literature than social services such as primary education. Comparative research examining the influence of cognitive biases on different governance levels remains scarce, further research could compare the likelihood of local, regional and national politicians to be prone to cognitive biases. Finally, this study suggests that future research could examine the influence of factors, such as time and resource availability, on politicians’ issue prioritisation decisions.

## Supplementary Material

NOC.docx

## Note

1. See Vis (2019) for a discussion on different traditions of heuristics in political decision-making.

## Appendix A. Descriptive statistics

**Table:**

	Politicians
**Gender** Female Male Other or unspecified	36.64% 62.66% .70%
**Age** Mean Standard deviation Minimum Maximum	51.71 12.85 21 83
**Education** No university degree University degree	18.36% 81.64%
**Tenure** Less than 1 year Between 1 and 5 years Between 6 and 10 years Between 11 and 15 years Between 16 and 20 years Between 21 and 25 years Between 26 and 30 years More than 30 years	2.94% 39.66% 22.15% 7.59% 9.14% 8.83% 4.57% 5.11%
**Region** Brussel Flanders Wallonia	6.97% 52.90% 40.12%
**n**	**1,291**

## Appendix B. Representativeness check

**Table:**

Variables	Population	Sample
**Gender**		
Female	38.96%	36.64%
Male	61.04%	62.66%
Other or unspecified	/	.70%
**Function**		
Executive function	26.80%	32.53%
Legislative function	73.20%	67.47%
**Region**		
Brussels	2.83%	6.97%
Flanders	56.78%	52.90%
Wallonia	40.39%	40.12%
**Population size**	20,327	21,994
**Population median income**	51,298	51,554
**Number of observations**	**13,035**	**1,291**

### Appendix C1. Balance checks (full sample; individual characteristics) – One-way analysis of variance (ANOVA)

**Table:**

	Experimental group	Means	N	p	Df
Female	Negative first (no justification)	.374	356	.789	3
	Positive first (no justification)	.358	344		
	Negative first (justification)	.349	298		
	Positive first (justification)	.386	293		
Male	Negative first (no justification)	.621	356	.822	3
	Positive first (no justification)	.637	344		
	Negative first (justification)	.641	298		
	Positive first (justification)	.608	293		
Age	Negative first (no justification)	51.42	356	.156	3
	Positive first (no justification)	52.77	344		
	Negative first (justification)	51.99	298		
	Positive first (justification)	50.51	293		
University diploma	Negative first (no justification)	.812	356	.990	3
	Positive first (no justification)	.817	344		
	Negative first (justification)	.822	298		
	Positive first (justification)	.816	293		

Note: one-way analysis of variance (ANOVA) to test whether they are significant differences between the four experimental groups with respect to participants’ gender, age, education, and region. * *p* < .05, ** *p* < .01, *** *p* < .001.

### Appendix C2. Appendix C Balance checks (full sample; municipal characteristics) – One-way analysis of variance (ANOVA)

**Table:**

	Experimental group	Means	N	p	Df
Brussels region	Negative first (no justification)	.076	356	.543	3
	Positive first (no justification)	.061	344		
	Negative first (justification)	.084	298		
	Positive first (justification)	.058	293		
Flanders region	Negative first (no justification)	.522	356	.882	3
	Positive first (no justification)	.541	344		
	Negative first (justification)	.513	298		
	Positive first (justification)	.539	293		
Wallonia region	Negative first (no justification)	.402	356	.999	3
	Positive first (no justification)	.398	344		
	Negative first (justification)	.403	298		
	Positive first (justification)	.403	293		
Population size	Negative first (no justification)	27,935	356	.066	3
	Positive first (no justification)	28,183	344		
	Negative first (justification)	37,536	298		
	Positive first (justification)	30,217	293		
Median income	Negative first (no justification)	51,341	356	.760	3
	Positive first (no justification)	51,654	344		
	Negative first (justification)	51,370	298		
	Positive first (justification)	51,831	293		

Note: one-way analysis of variance (ANOVA) to test whether they are significant differences between the four experimental groups with respect to participants’ gender, age, education, and region. * *p* < .05, ** *p* < .01, *** *p* < .001.

## Appendix D. OLS regression coefficient with attention to the evidence as outcome

**Table:**

Accountability (ref. no accountability)	11.34*** (.001)
Order of evidence (ref. positive first)	2.87 (.183)
Accountability*Order	−5.64 (.238)
Constant	38.15*** (.000)
Adjusted R^2^	.012
Number of observations	1,291

Note: OLS regression coefficients with p-values into parentheses. ^+^ *p* < .1, * *p* < .05, ** *p* < .01, *** *p* < .001.

## Appendix F. OLS regression coefficients (H_3_)

**Table:**

Accountability (ref. no accountability)	−.675 (.679)
Order of evidence (ref. positive first)	−1.418 (.346)
Accountability*Order	1.126 (.628)
Constant	62.65*** (.000)
Adjusted R^2^	.0007
Number of observations	1,291

Note: OLS regression coefficients with p-values into parentheses. ^+^ *p* < .1, * *p* < .05, ** *p* < .01, *** *p* < .001.

## Appendix G. Dropouts (full sample) – One-way analysis of variance (ANOVA)

**Table:**

	Experimental group	Means	N	p	Df
Dropout	Negative first (no justification)	.051	375	.000	3
	Positive first (no justification)	.063	367		
	Negative first (justification)	.205	375		
	Positive first (justification)	.206	369		

Note: one-way analysis of variance (ANOVA) to test whether they are significant differences between the four experimental groups with respect to participants’ dropout. ^+^ p < .1, * p < .05, ** p < .01, *** p < .001.
