# Supplementary material for: Issue prioritisation decisions by local politicians: the role of order effects and justification requirements
Source: Local Gov Stud. 2024 Jul 8;51(2):317–43. doi: 10.1080/03003930.2024.2374906 (PMC11969987; doi:10.1080/03003930.2024.2374906)
Supplement: NOC.docx [file FLGS_A_2374906_SM4013.docx]

Amandine Lerusse is an Assistant Professor at the Institute of Public Administration, Leiden University. Her research examines, through experimental approaches, politicians' and public managers' decision-making behavior and preferences when they use performance information.
